# Supportive actions towards people with mental health problems in the community: A national survey of Australian adults

**DOI:** 10.1177/00048674261421778

**Published:** 2026-02-16

**Authors:** Anthony F Jorm, Nicola J Reavley, Shurong Lu, Ellie Tsiamis, Amy J Morgan

**Affiliations:** Melbourne School of Population and Global Health, The University of Melbourne, Melbourne, VIC, Australia

**Keywords:** Mental health first aid, social support, suicide, Australia

## Abstract

**Background:**

People with mental health problems often report that they are avoided and discriminated against. However, less is known about the occurrence of supportive actions. This study aimed to investigate the prevalence in Australian adults of actions recommended by expert consensus mental health first aid guidelines, as well as actions not recommended.

**Methods:**

A national survey was carried out with 6045 Australians aged 18+ who were members of the *Life in Australia* probability-based online panel. Participants were asked about actions they intended to take to support a person with a mental health problem or in a mental health crisis. Those who had actually provided support to someone in the past 12 months were asked about what supportive actions they took, while those who had personally had a mental health problem were asked what support they had received.

**Results:**

Respondents more frequently reported providing actions that were recommended in mental health first aid guidelines than those not recommended (medians across actions of 88.2% vs 37.8%). However, people who had a mental health problem in the past 12 months reported actually receiving recommended actions less frequently (median across actions of 65.5%). Actions to support a person at suicide risk were particularly in need of improvement, with only 41.8% of suicidal persons being asked about suicidal thoughts.

**Conclusion:**

The findings show a need to further upskill Australian adults on how best to support people with mental health problems or in a mental health crisis, particularly those at suicide risk.

## Background

Stigma and discrimination towards people with mental illness have been recognized as a major issue globally, adding to the negative impacts of the illness itself (Thornicroft et al., 2022). A recent Australian national survey of people with mental health problems found that 67.7% agreed that stigma and discrimination were worse than the mental health problem itself (Reavley et al., 2025), indicating a need for continued action to reduce these.

A complementary goal to reducing stigma and discrimination is increasing positive support to people with a mental illness. The prevalence of mental illness is so common that everyone will have contact with someone affected. According to the 2020–2022 National Study of Mental Health and Wellbeing in Australia, 20% of people aged 16-85 years had a mental disorder in the previous 12 months (Slade et al., 2025), while another national survey found that 51% of Australian adults aged 18+ years reported knowing someone with a mental health problem in the past 12 months (Reavley et al., 2018). While the majority know someone with depression or anxiety problems, contact with people with other disorders and at suicide risk is not uncommon (Nicholas et al., 2019; Reavley et al., 2018). These statistics show that there is considerable potential to increase supportive interactions in the population.

One class of supportive behaviours has been termed ‘mental health first aid’ and defined as

*the help offered to a person developing a mental health problem, experiencing a worsening of an existing mental health problem or in a mental health crisis; the first aid is given until appropriate professional help is received or until the crisis resolves*. (Kitchener et al., 2015)

This definition distinguishes initial support provided by a member of the public from on-going support provided by someone in a carer role and from professional help.

Surveys in a number of countries have collected data on the intentions or actions of members of the public towards a person with a mental health problem. Open-ended responses about these intentions or actions have been scored according to how closely they adhere to a mental health first aid action plan (Kitchener et al., 2015). This plan involves: approach the person, assess and assist with any crisis; listen and communicate non-judgementally; give support and information; encourage the person to get appropriate professional help; and encourage other support. Using a scoring system with a 0–12 range, mean scores of <3 have been found with Australian adults (Jorm et al., 2019; Rossetto et al., 2014) and adolescents (Mason et al., 2015), Sri Lankan university students (Amarasuriya et al., 2017), British university students (Davies et al., 2016), Lithuanian university students (Čėnaitė et al., 2025) and Japanese older adults (Yoshioka and Rossetto, 2024), indicating considerable room for improvement.

Particular inadequacies have been identified in Australian adults’ helping intentions and actions towards persons with severe distress and experiencing suicidal thoughts, with only 48.5% asking the person if they had been thinking about killing themselves, 39.0% going to an appointment with a professional with the person and 19.2% calling a crisis line (Nicholas et al., 2019; Nicholas et al., 2020).

In order to find out specific mental health first aid actions that are likely to be helpful, a programme of research has been carried out to develop a series of guidelines for the public on how to best assist a person developing a mental health problem or in a mental health crisis (Jorm and Ross, 2018). These guidelines have been developed using the Delphi expert consensus method, with a consensus of both professional and lived-experience experts being required for a first aid action to be included in a guideline. These guidelines have been used to inform the curriculum content of Mental Health First Aid training courses, and to provide a standard against which to evaluate the quality of supportive behaviours in the community.

More recently, a new measure has been developed to assess the quality of mental health first aid behaviours, which does not require time-consuming scoring of open-ended responses. This Mental Health Support Scale (MHSS) was developed from a pool of actions that were recommended or not recommended in mental health first aid guidelines (Morgan et al., 2023). The items were selected from this pool using item response theory, which ensured that items were both good indicators of the underlying trait and discriminated across the full range of that trait. Versions of the MHSS were developed to measure intentions to provide support to a hypothetical person with a mental health problem, support actually provided to a person in the recent past and support received if the respondent had a mental health problem. Items asking about intended support can be answered by anyone, while those asking about provided or received support can only be answered by those with relevant experiences. The assessment of intentions as a proxy for actual behaviour is based on consistent evidence that intentions predict behaviour in general (Webb and Sheeran, 2006) as well as mental health first aid actions specifically (Rossetto et al., 2016; Usmani et al., 2023; Yap and Jorm, 2012).

While the MHSS is an advance over previous measures of supportive actions, it has not been used to assess the prevalence of such actions in a national population sample. Furthermore, no previous study has assessed the quality of both support provided and received as well as support intended. The aim of the current paper is to provide descriptive data on the prevalence of intended, provided and received supportive actions using the items from the MHSS in a national survey of Australian adults, and to identify areas where improvement is particularly needed.

## Methods

### Sample

The survey was carried out with the *Life in Australia* online panel established by the Social Research Centre (Kaczmirek et al., 2019). Members of this panel are recruited via random digit dialling or address-based sampling and have agreed to provide their contact details to take part in surveys on a regular basis. People cannot enrol unless invited to participate. The target population was adults aged 18+ years resident in Australia. A stratified random sample was drawn from the panel to come as close as possible to the Australian national population distribution on the stratification variables. The strata were age (18–34, 35–44, 55–64, 65+), gender, education (less than bachelor’s degree, bachelor’s degree or above) and speaking a language other than English at home. There were 7954 panellists invited for the current survey and 6045 of these completed it. The completion rate, defined as completed surveys as a percentage of all invited *Life in Australia* panellists, was 76%.

### Survey procedure

The Social Research Centre sent an initial survey invitation via email and SMS (where available), followed by multiple email reminders and a reminder SMS. Up to 5 reminders in different modes (including email, SMS and telephone) were administered within the fieldwork period. The survey was carried out in July 2025. Participants were offered an incentive valued at AUD 10 to participate.

### Survey questionnaire

The survey used a slightly modified version of the MHSS, which has questions about intentions to provide support to a person experiencing a mental health problem or crisis, provision of such support to a person in the last 12 months and receipt of such support in the past 12 months (Morgan et al., 2023). The validity of the MHSS-Intended and MHSS-Provided scales is indicated by discrimination between groups with and without mental health first aid expertise (Mental Health First Aid instructors vs the general public) (Morgan et al., 2023). The validity of the MHSS-Received scales is supported by a cross-sectional study of 1116 adults showing that receiving recommended support was associated with perceived benefits by the recipient, including improved mental health (*r* = 0.37) and a closer relationship with the helper (*r* = 0.39) (Morgan et al., 2025b).

Modifications to the MHSS involved three additional items about not-recommended actions in mental health first aid guidelines which were added by the authors to the original 23 items to broaden coverage in this area. These items were ‘*Made sure they heard the helper’s opinion and experiences*’, ‘*Tried to solve problems for them*’ and ‘*Told them they had to get better*’.

The questionnaire also included questions which are not used in the current paper, covering confidence in providing support, desire for social distance, barriers to providing help, medium of providing help, mental health first aid training experience, outcome of help, personal history of mental health problems or crises, and sociodemographic characteristics. Analyses of data from these questions will be the subject of subsequent publications on this study.

Prior to fieldwork, cognitive interviewing was conducted with the questionnaire to ensure that the instructions, questions and response options were clearly understood by respondents.

The text of the relevant questions is given in the Supplementary File, and abbreviated versions are given in Tables 1–4. Below is an overview of the instructions given to participants.

**Table 1.** Percent of respondents reporting that an action was intended, provided or received to support a person with a mental health problem.

Action	Intended Percent [95% CI] (*N* = 6024–6039)	Provided Percent [95% CI] (*N* = 1817–1823)	Received Percent [95% CI] (*N* = 768–818)
Asked whether had thoughts of harming themselves or others	62.0 [60.8, 63.2]	57.7 [55.4, 60.0]	35.0 [31.7, 38.3]
Discussed wishes about privacy and confidentiality	73.1 [72.0, 74.2]	64.9 [62.7, 67.1]	40.0 [36.6, 43.5]
Listened to problems and tried to provide solutions^a^	81.6 [80.7, 82.6]	Not asked	Not asked
Let know listening to what they are saying by restating and summarizing	84.7 [83.8, 85.6]	88.2 [86.6, 89.7]	71.8 [68.5, 74.9]
Communicated clearly and simply, and repeated things where necessary	88.2 [87.4, 89.0]	92.2 [90.9, 93.4]	80.1 [77.1, 82.8]
Made sure they heard helper’s opinion and experiences^a^	Not asked	64.3 [62.0, 66.5]	53.9 [50.4, 57.43]
Told them they have to get their act together^a^	14.0 [13.1, 14.8]	14.9 [13.3, 16.6]	22.9 [20.1, 25.9]
Tried to solve problems for them^a^	Not asked	34.1 [31.9, 36.3]	40.9 [37.5, 44.3]
Conveyed message of hope by telling them help is available and things can get better	89.4 [88.6, 90.1]	90.5 [89.1, 91.8]	84.0 [81.3, 86.4]
Tried to cheer them up by telling them that things don’t seem that bad^a^	35.0 [33.8, 36.2]	31.6 [29.5, 33.8]	44.3 [40.9, 47.7]
Told them they had to get better^a^	Not asked	26.0 [24.0, 28.1]	37.2 [33.9, 40.6]
Offered them information and resources appropriate to their situation	79.0 [77.9, 80.0]	78.6 [76.6, 80.5]	54.9 [51.4, 58.4]
Discussed their options for seeking professional help	88.3 [87.5, 89.1]	88.3 [86.7, 89.7]	74.8 [71.7, 77.7]
Asked whether they have other supportive people they can rely on	85.4 [84.5, 86.3]	82.1 [80.2, 83.8]	57.4 [53.9, 60.8]
Discussed with them whether they are interested in self-help strategies	68.6 [67.5, 69.8]	79.3 [77.4, 81.2]	65.5 [62.2, 68.8]

*N*s provided are unweighted.

a These actions are not recommended by experts.

**Table 2.** Percent of respondents reporting that an action was intended, provided or received to support a person who they suspect may be thinking about suicide.

Action	Intended Percent [95% CI] (*N* = 6024–6039)	Provided Percent [95% CI] (*N* = 916–918; and 367 where there was immediate risk)	Received Percent [95% CI] (*N* = 327–338; and 92–95 where there was immediate risk)
Asked if they have been thinking about suicide	63.5 [62.2, 64.7]	73.2 [70.1, 76.0]	41.8 [36.62, 47.2]
Told them how much it will hurt their family and friends if they were to kill themselves^a^	59.5 [58.3, 60.7]	61.4 [58.2, 64.6]	49.6 [44.3, 54.9]
Tried to make them understand that suicide is wrong^a^	45.3 [44.1, 46.6]	41.4 [38.1, 44.6]	34.7 [29.7, 40.0]
Asked if they have a plan for suicide – for example, how, when and where they intend to die	36.1 [34.9, 37.3]	43.6 [40.4, 46.9]	23.8 [19.5, 28.6]
(If the person at immediate risk) Encouraged them to get appropriate professional help as soon as possible	94.8 [94.2, 95.3]	93.0 [89.9, 95.3]	83.8 [75.6, 90.1]
(If the person at immediate risk) Made sure they are not left on their own	91.9 [91.2, 92.6]	91.1 [87.8, 93.8]	65.4 [55.4, 74.4]

*N*s provided are unweighted.

a These actions are not recommended by experts.

**Table 3.** Percent of respondents reporting that an action was intended, provided or received to support a person who is out of contact with reality.

Action	Intended Percent [95% CI] (*N* = 6024–6039)	Provided Percent [95% CI] (*N* = 501)	Received Percent [95% CI] (*N* = 58–66)
Acknowledged they might be frightened by what they are experiencing	89.5 [88.7, 90.2]	88.3 [85.3, 91.0]	72.9 [62.2, 82.0]
Tried to convince them that their beliefs and perceptions are false^a^	36.2 [35.0, 37.4]	46.8 [42.5, 51.2]	61.5 [49.8, 72.3]
Listened to them talk about their experiences even though you knew they are not based in reality	87.4 [86.5, 88.2]	94.8 [92.6, 96.6]	65.5 [54.6, 75.4]

*N*s provided are unweighted.

a These actions are not recommended by experts.

**Table 4.** Percent of respondents reporting that an action was intended, provided or received to support a person whose mental health problem is having a major impact on their life but they are reluctant to seek professional help.

Action	Intended Percent [95% CI] (*N* = 6024–6039)	Provided Percent [95% CI] (*N* = 1045)	Received Percent [95% CI] (*N* = 368–371)
Found out if there are specific reasons why they do not want to seek professional help	87.2 [86.4, 88.1]	76.2 [73.5, 78.7]	54.4 [49.4, 59.4]
Let them know they can contact you if they change their mind about seeking help	92.3 [91.6, 92.9]	92.9 [91.2, 94.4]	73.4 [68.7, 77.7]

*N*s provided are unweighted.

#### Support-intended questions

The lead-in for the support-intended questions was:

*For the purposes of this project, a ‘mental health problem’ occurs when a person feels depressed, anxious or emotionally stressed over a period of weeks or more, and this interferes with their life. It could include, for example, depression, anxiety disorders, eating disorders, substance use disorders, schizophrenia, bipolar disorder or personality disorders. Thinking about someone you know well, such as a family member, friend or colleague. Imagine this person is experiencing a mental health problem, experiencing the worsening of an existing mental health problem, or is in a mental health crisis (e.g. they are suicidal). How likely is it that you would take the following actions with the person?*

Next followed questions where the respondent was asked to imagine various scenarios and report how likely they were to take specific actions. The scenarios were: ‘*imagine you suspect that this person may be thinking about suicide*’, ‘*imagine this person is at immediate risk of suicide*’, ‘*imagine this person was out of contact with reality, for example, experiencing delusions, hallucinations, or paranoia*’ and ‘*imagine the person’s mental health problem is having a major impact on their life but they are reluctant to seek professional help*’.

Items were rated on the following scale: 1. Very unlikely, 2. Unlikely, 3. Neither likely nor unlikely, 4. Likely, 5 Very likely. The order of items within each topic was randomized.

#### Support-provided questions

The lead-in for the support-provided questions was: ‘*Has anyone you know well, such as a family member, friend or colleague, ever developed a mental health problem, or had a mental health crisis (e.g. they were suicidal)?*’ Response options were: Yes, in the last 12 months; Yes, but more than a year ago; No. If the respondent knew more than one person in the last 12 months, they were instructed to think about the most recent person. Several questions then followed about the characteristics of this person, e.g. relationship, age, gender. The participants were then asked: ‘*Over the last 12 months, did you try to help the person with this problem*’. Then followed some questions about any barriers to providing help.

Next the participant was asked: ‘*Did you do any of the following to try to support the person?*’ and given the same scenarios as for support intended. Again, response options for these questions were: Yes, No, Don’t know/Unsure, Refused/Prefer not to say. The order of items within each topic was again randomized.

#### Support-received questions

The lead-in for the support-received questions was: ‘*Have you ever experienced a mental health problem, or had a mental health crisis (e.g. you were suicidal)?*’ Options were: Yes, in the last 12 months; Yes, but more than a year ago; No. Following a question about the nature of the problem, participants who experienced a problem in the last 12 months were asked: ‘*Did anyone you know well, such as a family member, friend or colleague, try to support you with your problem?*’. This was followed by questions about the characteristics of the helper, and then:

*Take a moment to reflect on your experience of receiving support from this person. In the next section you will be presented with statements of actions that the person may have used. Please reflect carefully on your experience when responding to these. Reflecting on your experience of receiving support from this person. Did they do any of the following to try to support you?*

The questions then followed the same scenarios as for supported intended and provided.

Response options for these questions were: Yes, No, Don’t know/Unsure, Refused/Prefer not to say. The order of items within each topic was again randomized.

### Statistical analysis

The percent prevalence in the sample was calculated for endorsing each question concerning support intended, received and provided. The responses to the questions on support intended were dichotomized to make them comparable with the dichotomous responses to the support provided and received items. An endorsement was coded 1 if the participant responded ‘Likely’ or ‘Very likely’ and 0 otherwise. For the questions on support provided, an endorsement was ‘Yes’ among participants who knew someone developing a mental health problem or having a mental health crisis in the last 12 months. For the questions on support received, an endorsement was ‘Yes’ among participants who had experienced a mental health problem or had a mental health crisis in the last 12 months. Responses of Don’t know/Unsure and Refused/Prefer not to say were very low in frequency and coded as missing data.

The Clopper-Pearson (Exact) method was used to calculate 95% confidence intervals of prevalence. Sample weights were used to reduce any biases in the sample compared to the Australian population aged 18 years or older. The characteristics used for adjusting weights were number of adults in the household, age group by highest education, gender, language other than English spoken at home, geographic location (capital vs rest of state) and state or territory of residence. Differences between prevalences were interpreted as significant where there were non-overlapping confidence intervals.

With items as the unit of analysis, Mann–Whitney tests were used to compare the percent endorsement of recommended and non-recommended items, and Friedman’s analyses of variance by ranks to compare the percent endorsement of intended, provided and received items. The *p* < 0.05 level was used for these comparisons.

All analyses were carried out using SPSS version 30.0.0.0.

## Results

The sample consisted of 6045 individuals with the following sociodemographic breakdown: female 59.2%, aged 18–24 years 5.7%, 25–34 years 16.2%, 35–44 years 21.4%, 45–54 years 17.2%, 55–64 years 16.8%, 65–74 years 14.7%, 75+ years 8.0%, Australian born 71.1%, use of a language other than English at home 17.1%, residence in capital city 68.5%, most socio-economically disadvantaged quintile 14.6%, least disadvantaged quintile 22.9% and Bachelor’s degree or higher 44.4%.

In the weighted sample, the prevalence of knowing someone with a mental health problem over the past 12 months was 31.8% [95% CI: 30.6, 33.0]. Of those who knew someone, 92.4% [95% CI: 91.2, 93.6] tried to help. The prevalence of reporting having a personal mental problem was 19.7% [95% CI: 18.7, 20.7]. Of those who had a problem, 72.0% [95% CI: 69.4, 74.5] reported that someone tried to support them.

Table 1 shows the percent of respondents reporting that an action was intended, provided or received to support a person with a mental health problem. Table 2 shows the percent reporting actions to support a person who may be thinking about suicide, Table 3 the percent reporting actions to support a person out of contact with reality, and Table 4 the percent reporting actions to support a person who is reluctant to seek professional help. The unweighted *N*s reported in these tables vary slightly between analyses because of missing data on relevant questions.

**Figure 1.**
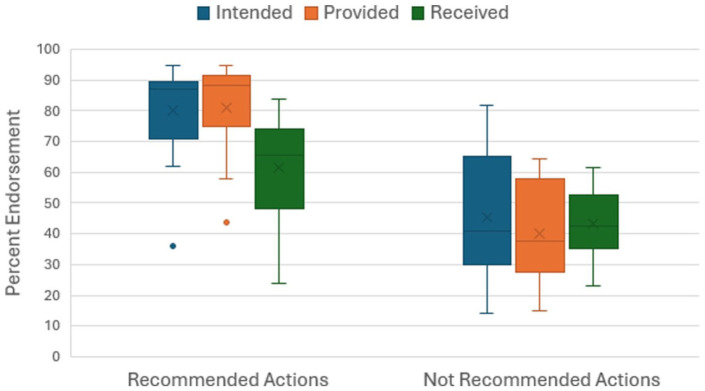
Percent endorsement across recommended and not-recommended actions.

Figure 1 gives a broad-brush summary of the findings in these tables. It shows a box and whisker plot of percent endorsement across recommended and not-recommended actions for support intended, provided and received. For recommended actions, the median endorsement was 87.2% for intended and 88.2% for provided actions, but only 65.5% for received actions. For the not-recommended actions, the median endorsement was similar for intended (40.8%), provided (37.8%) and received actions (42.6%). Mann–Whitney tests showed that recommended actions had a higher percent endorsement than non-recommended actions for intended, provided and received items, with *p*-values of 0.002, <0.001 and 0.011, respectively. For recommended actions, Friedman tests showed that both intended and provided actions had a higher percent endorsement than received actions (both *p* < 0.001). However, there were no significant differences between non-recommended intended, provided and received actions.

The following recommended actions were the most commonly intended, provided and received (over 70% for each version of the questionnaire): letting the person know that the helper is listening to what they are saying by restating and summarizing; communicating clearly and simply, and repeating things where necessary; conveying a message of hope by telling the person that help is available and things can get better; discussing their options for seeking professional help; if the person is at suicide risk, encouraging them to get appropriate professional help as soon as possible; if the person is out of contact with reality, acknowledging that they may be frightened by what they are experiencing; and if the person is reluctant to seek professional help, letting them know that they can contact the helper if they change their mind about seeking help.

The most commonly reported not-recommended actions (over 30% for each version of the questionnaire) were: trying to find solutions for the person; making sure the person hears the helper’s opinion and experiences; trying to cheer them up by telling them that things don’t seem that bad; trying to make a suicidal person understand that suicide is wrong, and telling them how much it will hurt their family and friends it they were to kill themselves; and trying to convince a person who is out of contact with reality that their beliefs and perceptions are false.

## Discussion

It is encouraging that actions that are recommended by experts were in general reported more frequently than those not recommended. However, there is considerable room for improvement, with substantial minorities not providing recommended support and taking actions that are not recommended by experts.

A striking finding from the survey is that actions recommended by experts were much less likely to be reported as received (median across actions of 65.5%) than as intended or provided (medians across actions of 87.2% and 88.2%, respectively). On the other hand, actions not recommended by experts were similar in prevalence for intended, provided and received support (median across actions of 40.8%, 37.8% and 42.6%, respectively). One interpretation of this finding is that people often over-estimate the quality of support they provide or intend to provide. However, in making a direct comparison between the responses on support intended and provided and the responses on support received, it must be acknowledged that respondents are not reporting on the same interactions. There may be selection factors in thinking about a recent person who was supported that favour more positive interactions. Studies of dyads (e.g. members of the same family where one is the supporter and the other the recipient) are needed to confirm this. Furthermore, the analysis of intended actions grouped together ‘Very likely’ and ‘Likely’ responses, with the prevalences being lower if only ‘Very likely’ responses are counted. Nevertheless, the perspectives of people who received support may provide a more relevant and accurate indication of the quality of supportive actions in the community. If this is the case, then the findings on support received indicate an even greater need to improve supportive actions in the Australian population.

One area where there is a particular need for improvement is in supporting a person at suicide risk. There was a reluctance of many people to talk openly about suicidal thoughts and to ask about plans for suicide. Furthermore, the use of guilt-inducing strategies to prevent suicide (‘*Told them how much it will hurt their family and friends if they were to kill themselves*’ and ‘*Tried to make them understand that suicide is wrong*’) were common (provided by 61.4% and 41.4%, respectively). These strategies may dissuade a suicidal person from talking openly with a potential helper in the future. Some of these findings are consistent with a 2017 Australian national survey which found that many people were reluctant to talk openly about suicide and ask questions about suicide risk (Nicholas et al., 2019; Nicholas et al., 2020). Direct comparison between the earlier survey and the present one is difficult because of differences in methodology, e.g. the earlier survey presented vignettes of persons in severe distress and asked respondents whether they had supported someone with a similar problem in the last 12 months. However, there is some evidence that there may have been an improvement. In the 2017 survey, 48.5% [95% CI: 44.4, 52.6] ‘*asked whether they had been thinking about killing themselves*’, while the current survey found that 57.7% [95% CI: 55.4, 60.0] asked whether the person ‘*had thoughts of harming themselves or others*’ and, if they suspected the person may be thinking about suicide, 73.2% [95% CI: 70.1, 76.0] ‘*asked if they had been thinking about suicide*’. Given the recommendations in the Australian National Suicide Prevention Strategy 2025–2035 about the importance of reducing suicide stigma and connecting people who experience suicidal distress with supports (National Suicide Prevention Office, 2025), there is a need for continuing national monitoring of progress in this area.

Training in how to provide support can be effective. A recent cluster randomized controlled trial with Australian older men showed that suicide prevention training had positive effects on helping intentions and actual help provided and reduced stigmatizing attitudes at 7-month follow-up (Morgan et al., 2025). Similarly, a cluster randomized trial of teen Mental Health First Aid training of high school students showed improvements in intentions to support a peer with suicidal thoughts that were sustained at 12-month follow-up (Hart et al., 2020).

Strengths of the study are that it was based on an online panel recruited and weighted to match the sociodemographic breakdown of the Australian adult population and that 76% of panellists approached agreed to participate. Another strength is that the study asked about support for people with mental health problems from multiple perspectives. The major limitation is that the sample could have unknown biases due to greater participation by panellists who have a particular interest in mental health. If this were the case, then the estimates of support provided may be optimistic. A relevant indicator of participants’ interest in mental health is the prevalence of reporting having a personal mental problem. This was 19.7% [95% CI: 18.7, 20.7], which is similar to the 12-month mental disorder prevalence of 20% in the 2020–2022 National Study of Mental Health and Wellbeing in Australia (Slade et al., 2025). However, this similarity in prevalence must be viewed cautiously, as the methods involved in ascertaining cases were quite different.

## Conclusion

This national survey has shown areas where supportive actions towards people with mental health problems or in a mental health crisis need to be improved. Improvement is particularly needed in support for people who are suicidal. The findings can be used to guide the further development and roll out of interventions to improve support in the population and as a baseline for ongoing monitoring of progress in the area. We plan to provide more detailed analyses of factors associated with quality of support, including the effect of training, in subsequent papers.

## Supplemental Material

sj-docx-1-anp-10.1177_00048674261421778 – Supplemental material for Supportive actions towards people with mental health problems in the community: A national survey of Australian adults Supplemental material, sj-docx-1-anp-10.1177_00048674261421778 for Supportive actions towards people with mental health problems in the community: A national survey of Australian adults by Anthony F Jorm, Nicola J Reavley, Shurong Lu, Ellie Tsiamis and Amy J Morgan in Australian & New Zealand Journal of Psychiatry
