# Supplementary material for: Supportive actions towards people with mental health problems in the community: A national survey of Australian adults
Source: Aust N Z J Psychiatry. 2026 Feb 16;60(5):476–84. doi: 10.1177/00048674261421778 (PMC13109600; doi:10.1177/00048674261421778)
Supplement: sj-docx-1-anp-10.1177_00048674261421778 – Supplemental material for Supportive actions towards people with mental health problems in the community: A national survey of Australian adults [file sj-docx-1-anp-10.1177_00048674261421778.docx]

Survey questionnaire

The survey used a slightly modified version of the Mental Health Support Scale, which has questions about intentions to provide support to a person experiencing a mental health problem or crisis, provision of such support to a person in the last 12 months, and receipt of such support in the past 12 months (Morgan et al., 2023). The modifications involved some additional items about not-recommended actions.

The questionnaire also included questions which are not used in the current paper, covering confidence in providing support, social distance, barriers to providing help, medium of providing help, mental health first aid training experience, outcome of help, personal history of mental health problems or crises, and sociodemographic characteristics. The text of the relevant questions is given below. Questions marked with an asterisk are not recommended by experts.

*Support intended questions*

“For the purposes of this project, a ‘mental health problem’ occurs when a person feels depressed, anxious or emotionally stressed over a period of weeks or more, and this interferes with their life. It could include, for example, depression, anxiety disorders, eating disorders, substance use disorders, schizophrenia, bipolar disorder or personality disorders. Thinking about someone you know well, such as a family member, friend or colleague. Imagine this person is experiencing a mental health problem, experiencing the worsening of an existing mental health problem, or is in a mental health crisis (e.g. they are suicidal). How likely is it that you would take the following actions with the person?

a) Ask if they have been having thoughts of harming themselves or others

b) Discuss with them their wishes about privacy and confidentiality

c) Listen to their problems and try to provide solutions*

d) Let them know you are listening to what they are saying by restating and summarising what they have said

e) Communicate clearly and simply, and repeat things where necessary

f) Tell them they have to get their act together*

g) Convey a message of hope by telling them help is available and things can get better

h) Try to cheer them up by telling them that things don't seem that bad*

i) Offer them information and resources appropriate to their situation

j) Discuss their options for seeking professional help

k) Ask whether they have other supportive people they can rely on

l) Discuss with them whether they are interested in self-help strategies

For the next few questions, imagine you suspect that this person may be thinking about suicide. How likely is it that you would take the following actions with the person?

 a) Ask if they have been thinking about suicide

b) Tell them how much it will hurt their family and friends if they were to kill themselves*

c) Try to make them understand that suicide is wrong*

d) Ask if they have a plan for suicide – for example, how, when and where they intend to die

Imagine this person is at immediate risk of suicide. How likely is it that you would take the following actions?

a) Encourage them to get appropriate professional help as soon as possible – for example, see a mental health professional or someone at a mental health service

b) Make sure they are not left on their own

For the next few questions, imagine this person was out of contact with reality, for example, experiencing delusions, hallucinations, or paranoia. How likely is it that you would take the following actions with the person?

a) Acknowledge they might be frightened by what they are experiencing

b) Try to convince them that their beliefs and perceptions are false*

c) Listen to them talk about their experiences even though you know they are not based in reality

Imagine the person’s mental health problem is having a major impact on their life but they are reluctant to seek professional help. How likely is it that you would take the following actions?

a) Find out if there are specific reasons why they do not want to seek professional help

b) Let them know they can contact you if they change their mind about seeking help”.

Items were rated on the following scale: 1. Very unlikely, 2. Unlikely, 3. Neither likely nor unlikely, 4. Likely, 5 Very likely. The order of items within each topic was randomized.

*Support provided questions*

“Has anyone you know well, such as a family member, friend or colleague, ever developed a mental health problem, or had a mental health crisis (e.g. they were suicidal)?” Response options were: Yes, in the last 12 months; Yes, but more than a year ago; No. If the respondent knew more than one person in the last 12 months, they were instructed to think about the most recent person. Several questions then followed about the characteristics of this person, e.g. relationship, age, gender. The participants were then asked: “Over the last 12 months, did you try to help the person with this problem”. Then followed some questions about any barriers to providing help.

Next the participant was asked: “Did you do any of the following to try to support than person?

a) Asked them whether they had thoughts of harming themselves or others

b) Discussed with them their wishes about privacy and confidentiality

c) Let them know you were listening to what they were saying by restating and summarising what they have said

d) Communicated clearly and simply, and repeated things where necessary

e) Made sure they heard your opinion and experiences*

f) Told them they had to get their act together*

g) Tried to solve their problems for them*

h) Conveyed a message of hope by telling them help is available and things can get better

i) Tried to cheer them up by telling them that things don't seem that bad*

j) Told them they had to get better*

k) Offered them information and resources appropriate to their situation

l) Discussed their options for seeking professional help

m) Asked whether they had other supportive people they could rely on

n) Discussed with them whether they were interested in self-help strategies (e.g., regular exercise or meditation).

When you were supporting that person, did you even find out or suspect they might be thinking about suicide? [If yes] Did you do any of the following?

a) Asked if they had been thinking about suicide

b) Told them how much it would hurt their family and friends if they were to kill themselves*

c) Tried to make them understand that suicide is wrong*

d) Asked them if they had a plan for suicide - for example, how, when and where they intended to die

Did you ever think the person was at immediate risk of suicide? [If yes] Did you do any of the following?

a) Encouraged them to get appropriate professional help as soon as possible – for example, see a mental health professional or someone at a mental health service

b) Made sure they were not left on their own

When you were supporting that person, did you ever find out or suspect they might be out of contact with reality, for example, experiencing delusions (false beliefs), hallucinations (seeing or hearing things that aren’t real), or paranoia? [If yes] Did you do any of the following?

a) Acknowledged they might be frightened by what they were experiencing

b) Tried to convince them that their beliefs and perceptions were false*

c) Listened to them talk about their experiences even though you knew they were not based in reality

When you were supporting that person, were they ever reluctant to seek professional help even though their mental health problem was having a major impact on their life? [If yes] Did you do any of the following?

a) Found out if there were specific reasons why they did not want to seek professional help

b) Let them know they could contact you if they changed their mind about seeking help”.

Response options for these questions were: Yes, No, Don’t know/Unsure, Refused/Prefer not to say. The order of items within each topic was randomized.

*Support Received questions*

“Have you ever experienced a mental health problem, or had a mental health crisis (e.g. you were suicidal)?” Options were: Yes, in the last 12 months; Yes, but more than a year ago; No. Following a question about the nature of the problem, participants who experienced a problem in the last 12 months were asked: “Did anyone you know well, such as a family member, friend or colleague, try to support you with your problem?”. This was followed by questions about the characteristics of the helper, and then: “Take a moment to reflect on your experience of receiving support from this person. In the next section you will be presented with statements of actions that the person may have used. Please reflect carefully on your experience when responding to these. Reflecting on your experience of receiving support from this person. Did they do any of the following to try to support you?

a) Asked whether you had thoughts of harming yourself or others

b) Discussed with you your wishes about privacy and confidentiality

c) Tried to solve your problems for you*

d) Let you know they were listening to what you were saying by restating and summarising what you had said

e) Communicated clearly and simply, and repeated things where necessary

f) Made you listen to their opinion and experiences*

g) Told you that you had to get your act together*

h) Conveyed a message of hope by telling you that help is available and things can get better

i) Tried to cheer you up by telling you that things don't seem that bad*

j) Told you that you have to get better*

k) Offered you information and resources appropriate to your situation

l) Discussed with you your options for seeking professional help

m) Asked whether you had other supportive people you could rely on

n) Discussed with you whether you were interested in self-help strategies (e.g., regular exercise or meditation)

Did you have thoughts of suicide? [If yes] Did they do any of the following to try to support you?

a) Asked if you had been thinking about suicide

b) Told you how much it would hurt your family and friends if you were to kill yourself*

c) Tried to make you understand that suicide is wrong*

d) Asked if you had a plan for suicide – for example, how, when and whether you intend to die

Were you at immediate risk of suicide, for example, had a specific plan to suicide, means to carry out the plan, or an intention to do it? [If yes] Did they do any of the following to try to support you?

a) Encouraged you to get appropriate professional help as soon as possible – for example, see a mental health professional or someone at a mental health service

b) Made sure you were not left on your own

Were you out of contact with reality, for example, experiencing delusions (fixed false beliefs), hallucinations (seeing or hearing things that weren’t real), or paranoia? [If yes] Did they do any of the following to try to support you?

a) Acknowledged that you might be frightened by what you were experiencing

b) Tried to convince you that your beliefs and perceptions were false*

c) Listened to you talk about your experiences even though they were not based in reality

Were you reluctant to seek professional help? [If yes] Did they do any of the following to try to support you?

a) Found out if there were specific reasons why you did not want to seek professional help

b) Let you know you could contact them if you changed your mind about seeking help”.

Response options for these questions were: Yes, No, Don’t know/Unsure, Refused/Prefer not to say. The order of items within each topic was randomized.
